# Temporal Pattern of CABG and PCI after Non-ST Elevation Myocardial Infarction Among Elderly Patients from NHDS

**DOI:** 10.7759/cureus.6814

**Published:** 2020-01-29

**Authors:** Muhammad U Siddiqui, Codruta Chiuzan, Muhammad Danial Siddiqui, Syed Shayan Ali, Zunaira Naeem, Shariful Islam

**Affiliations:** 1 Hospital Medicine/Internal Medicine, Marshfield Clinic Medical Center, Rice Lake, USA; 2 Biostatistician, Mailman School of Public Health, New York City, USA; 3 Internal Medicine, University of South Dakota, Sioux Falls, USA; 4 Emergency Medicine, Yale University, New Haven, USA; 5 Pathology, Thomas Jefferson University, Philadelphia, USA; 6 Epidemiology and Public Health, Deakin University, Victoria, AUS

**Keywords:** non-st elevation myocardial infarction, percutaneous coronary intervention, coronary artery bypass surgery

## Abstract

Background

Management of elderly patients with Non-ST Elevation Myocardial Infarction (NSTEMI) continues to be a source of controversy due to underrepresentation in large-scale clinical trials and the increased risk of adverse outcomes after both invasive (Percutaneous coronary intervention and Coronary artery bypass grafting) and non-invasive therapies. Recent randomized clinical trials have shown improved short term and intermediate term outcomes among high risk NSTEMI patients receiving early invasive management versus conservative medical management. However, how this is reflected in U.S. clinical practice for elderly patients has not been reported.

Objective

To identify the trend of invasive management in patients with NSTEMI, particularly among elderly population.

Methods

We used data from National Hospital Discharge Survey to identify all adult patients with an International Classification of Diseases, Ninth Revision, Clinical Modification (ICD-9-CM) code for NSTEMI from the years 2005 to 2009. The goal was to investigate the trends in time of invasive therapy for patients diagnosed with NSTEMI. We then stratified the patients according to age >65 and ≤65, and compared the temporal trends between two age groups.

Results

Among 21,306 patients diagnosed with NSTEMI between 2005 and 2009, the median age was 73 years (IQR: 61-82 years), 54% were males and 57% were White. The proportions of patients age>65 years receiving invasive management (21%, N=13978) was significantly lower than those age≤65 (41%, N=7328) (p<0.001). Moreover, in both age groups, the proportion of patients receiving early invasive management decreased substantially over time (p<0.001).

Conclusion

Despite numerous studies promoting the use of early invasive management for NSTEMI patients, the proportion of patients receiving invasive intervention gradually decreased from 2005-2009, more so in elderly population. The decrease seen in overall proportion of patients receiving invasive therapy could be associated with older median age of NSTEMI patients; 73 years (IQR: 61-82). Our future analyses will investigate if this trend maintains after adjusting for other factors (sex, co-morbid conditions, insurance status, year of procedure, hospital region, and hospital bed-size) thought to be associated with the management of NSTEMI in elderly patients.

## Introduction

Acute coronary syndrome can present as unstable angina (UA), non-ST elevation myocardial infarction (NSTEMI) or ST elevation myocardial infarction (STEMI). In most situations, UA/NSTEMI are caused by coronary artery disease (CAD) leading to an increased risk of cardiac death and myocardial infarction [1]. Prompt revascularization is the therapy of choice in STEMI, however clear guidelines are lacking to address NSTEMI [2]. As per the American College of Cardiology/American Heart Association guidelines, two treatment pathways have emerged for treating patients with UA/NSTEMI. The rationale to choose between an invasive and a conservative (ischemia-guided) strategy is a much debatable one and especially so in elderly population. The decision in most cases is left to physician’s evaluation of the risk for an adverse outcome versus benefit for a conservative versus initial invasive treatment [1].

The conservative or ischemia- guided strategy is applied unless the risk of patient experiencing refractory or recurrent ischemic symptoms or developing hemodynamic instability is high. In this treatment, non-invasive evaluation to detect severe ischemia must be incorporated. This may include a stress test before discharge. Antiplatelet agents are also recommended to prevent adverse outcomes. The main advantage with this strategy is that many patients stabilize on this treatment, reducing the use of cardiac catheterization and avoidance of costly and unnecessary invasive procedures. Conservative strategy is indicated and can be safely used with a low score on scales like TIMI or GRACE, or absence of high risk as per physician judgment [1].

Invasive strategy is necessitated when patient experiences recurrent angina or ischemia at rest or with low level activities despite medical therapy, when cardiac biomarkers are elevated, when there are signs and symptoms of heart failure or when there is hemodynamic instability. Other factors include sustained ventricular tachycardia, prior percutaneous coronary intervention (PCI) or coronary artery bypass graft surgery (CABG), identification of high risk with non-invasive testing or reduced LV function [1]. An invasive approach requiring the use of angiography facilitates with risk stratification. Appropriate identification of left ventricle (LV) dysfunction or left main CAD may prompt timely coronary artery bypass graft surgery (CABG) and percutaneous coronary intervention (PCI) facilitating good eventual prognosis [1].

Despite several recommendations, the treatment trend for NSTEMI is not well established more so in elderly population. This subgroup of patients, which is an increasing proportion of those with acute coronary syndrome [3], has largely remained underrepresented in randomized clinical trials and no set guidelines have been formulated [4]. The subgroup of elderly population is much different from other subgroups owing especially to existence of multiple co-morbidities in these patients, polypharmacy and occurrence of adverse and often rare side-effects to different drugs including antithrombolytics [5]. The focus of our study is to determine the trend of conservative versus invasive treatment regimen for NSTEMI and especially so in elderly population.

## Materials and methods

We collected data from the National Hospital Discharge Survey (NHDS) database. National Center for Health Statistics conducts a study annually on hospitalized patients called National Hospital Discharge Survey (NHDS). They collect information on characteristics of inpatients discharged from over 500 non-federal short-stay hospitals. Data characteristics consist of age, sex, and hospital geographic location for every patient. It also contains 7 diagnostic codes and 4 procedural codes using the International Classification of Disease, 9th revision, clinical modification (ICD-9-CM).

National Hospital Discharge Survey data from 2005 - 2009 was obtained. We searched for all the patients from 2005-2009 who had ICD-9-CM diagnosis of NSTEMI (410.7). We stratified the patients diagnosed with NSTEMI in two groups: age<65 and age>65 years. Among these patients, we identified those who had a procedure code of percutaneous coronary intervention (V45.82) or coronary artery bypass (V45.81). To account for the possibility that the invasive management was not specifically for NSTEMI, we excluded all patients who had diagnostic codes for STEMI (410.9) and heart valve replacement (V43.3).

Statistical analysis

Descriptive statistics were used to summarize patients’ characteristics. Continuous variables were reported as medians (interquartile range, IQR) and categorical variables were expressed as proportions (%). Cochran-Armitage trend test was used to measure the decrease in the proportion of patients receiving early invasive management between two age categories: age ≤ 65 and age > 65. All tests employed a type I error (alpha) set at 0.05. Statistical analyses were carried out using SAS (version 9.4) software (Cary, North Carolina).

## Results

From 2005-2009 we identified 21,306 hospitalizations in the NHDS with a diagnosis of NSTEMI, of which 5,939 had a procedure code for angiography or coronary artery bypass surgery (invasive management). 65.60% were greater than 65 years of age and 54% of the patients were male (Table 1).

**Table 1:** Baseline characteristics of the study population

Patient Characteristics		
Age	<65	>65
	7,328	13,978
Gender	Male	Female
	11,505	9,801
Race	White	Others
	12,144	9162

**Figure 1:**
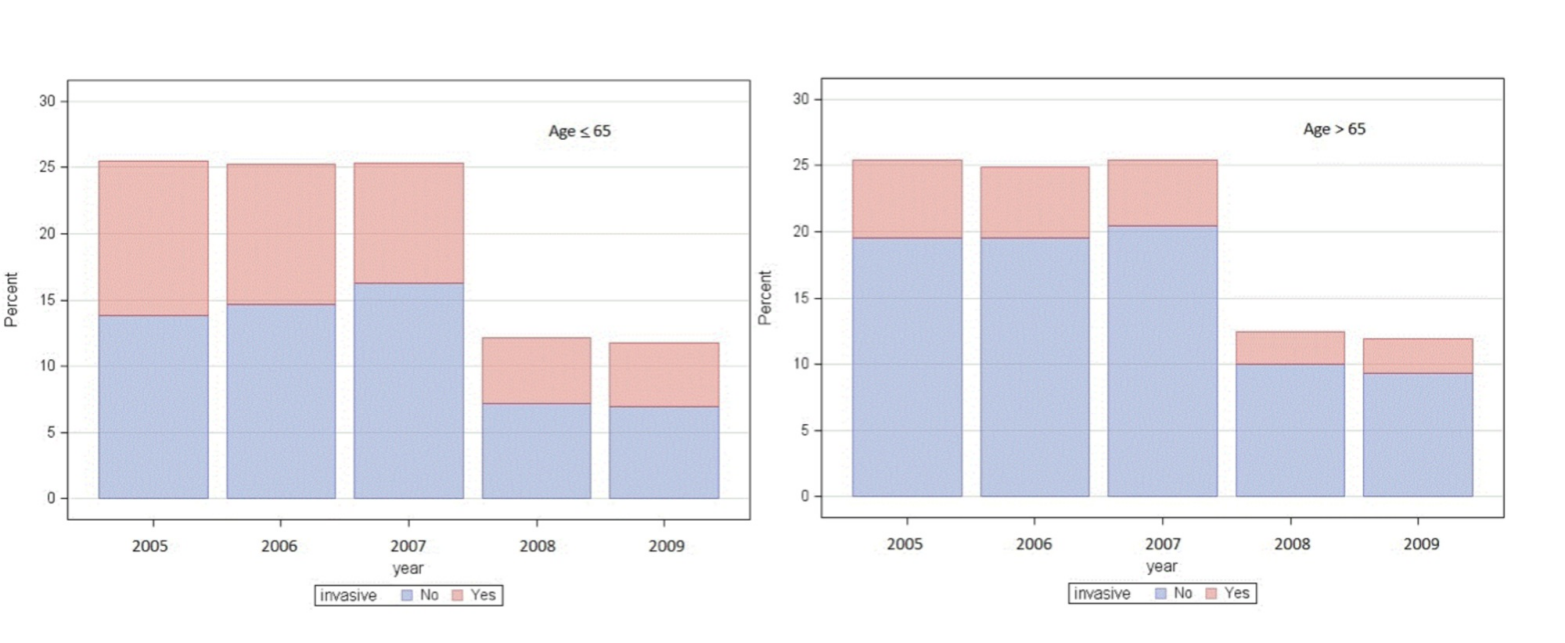
Graphic illustration of the time trend in the two age groups. The Y-axis shows the percent (relative frequency) out of the total of 5 years (N=7328 age≤65, and N=13978 age>65)

Between the years 2005-2009, the number of patients undergoing invasive management decreased substantially (p<0.001) (Figure 1). Moreover, patients with age >65 years had significantly lower invasive management for NSTEMI compared to patients of age <65 years undergoing invasive management for NSTEMI. Of 13,978 patients with age >65 years, 21% had invasive management. This was significantly lower when compared to 41% of 7,328 patients with age < 65 who underwent invasive management (p<0.001) (Figure 1).

## Discussion

This study demonstrated that invasive therapy for NSTEMI was more common in patients who are less than 65 years of age. In addition, a decreasing trend of invasive therapy was observed in both younger and older subgroups. These results differed from a similar study conducted in Italian Cardiac Units analyzing elderly population with NSTEMI over a course of 9 years. The use of an invasive approach rose from 26% in 2001 to 68.4% in 2010. Thirty-day mortality also fell down but not as drastically as the rise in administration of invasive therapy to these patients [4]. In Denmark, another similar study showed a rise in coronary angiography or percutaneous coronary intervention in patients with NSTEMI. However, a decline was seen in the use of CABG for the same [6].

Angeli et al. compared the benefit of early invasive to selectively invasive strategy. The endpoint of developing an MI or occurrence of death was found to be lower in early invasive strategy and the benefit was seen to be equal in both sexes [2]. The benefits from an early invasive strategy comes mainly as a result of improvement in medical therapy and antiplatelet strategies which have brought down the increased risk of bleeding. However, in patients older than 75 years old, bleeding events following PCI are still very high as reported by the TACTICS TIMI-18 trial. This again points to careful assessment of risk versus benefits by the physician based on individual patient’s comorbidities, previous complications, current medications, and also patient’s wishes. The age cut-off should never be the sole criterion [2].

Various clinical trials have been completed in an attempt to formulate clear guidelines regarding invasive or conservative management with mixed results [7-10]. TIMI IIIB recruited 1473 patients and followed them with either of the two treatment strategies. The study concluded that no significant difference existed in the rates of death and non-fatal MI between the two. However, the study found a significant reduction in hospital stay and in the rate of re-hospitalization with invasive strategy. The study however had its limitation in that a high crossover occurred to invasive therapy from the conservative group [10]. The VANQWISH trial also depicted similar results with no apparent benefit from the invasive approach. In fact, the study showed that the incidence of death and non-fatal MI was more in the invasive group [11].

The British Heart Foundation RITA 3 conducted a robust randomized trial to prove that interventional strategy is better than conservative one. The mean age of participants in this trial was 62 years. The study concluded that interventional strategy was able to reduce the risk of refractory or severe angina by half and with no increased risk of death or myocardial infarction [12]. RITA-3 trial established that an invasive over conservative treatment continued to provide benefit even after the first year and especially in high risk quartiles including elderly with multiple comorbidities like diabetes mellitus and renal dysfunction [4].

Selected elderly patients may benefit more with an invasive strategy as compared to younger patients albeit with an increased risk of bleeding complications. TACTICS-TIMI 18 looked at the benefits of invasive versus conservative strategy based on age groupings. Among patients aged more than 65 years, an early invasive strategy reduced the risk of death or MI at 30 days and six-months both. And at the age greater than 75, these benefits were seen to be more marked but with an increased incidence of major bleeding events [13].

This study had certain limitations. First, the NHDS data from 2010 onwards was not available when this study was conducted. Further data may show a change in the trend from what is observed in this study. Second, the number of hospitals participating in the NHDS repository decreased from 2008 onwards, leading to decreased number of patients with the disease of interest. Third, there is a risk of performance bias given retrospective nature of this study. Lastly, baseline characteristics of the two groups were not assessed which may have caused confounding.

## Conclusions

Our study indicated that the use of invasive management strategy is less utilized in elderly population than in younger population and over years there has been a decreasing trend in both age groups. This drop could be associated with high median age of patients in our study. There are still no clear recommendations on the use of one strategy over the other and prior studies have shown mixed outcomes of the two treatment protocols. More randomized controlled studies are needed to evaluate the role of these two approaches especially in the elderly population and formalization of proper guidelines. Our future analyses will investigate if this decreasing trend in use of invasive therapy continues after adjusting for other factors (sex, co-morbid conditions, insurance status, year of procedure, hospital region, and hospital bed-size) which are known to be associated with the management of NSTEMI in elderly patients.
